# phiC31 Integrase-Mediated Site-Specific Recombination in Barley

**DOI:** 10.1371/journal.pone.0045353

**Published:** 2012-09-14

**Authors:** Eszter Kapusi, Katja Kempe, Myroslava Rubtsova, Jochen Kumlehn, Mario Gils

**Affiliations:** Leibniz Institute of Plant Genetics and Crop Plant Research (IPK) Gatersleben, Gatersleben, Germany; Instituto Valenciano De Investigaciones Agrarias, Spain

## Abstract

The *Streptomyces* phage phiC31 integrase was tested for its feasibility in excising transgenes from the barley genome through site-specific recombination. We produced transgenic barley plants expressing an active phiC31 integrase and crossed them with transgenic barley plants carrying a target locus for recombination. The target sequence involves a reporter gene encoding green fluorescent protein (GFP), which is flanked by the *attB* and *attP* recognition sites for the phiC31 integrase. This sequence disruptively separates a *gusA* coding sequence from an upstream rice actin promoter. We succeeded in producing site-specific recombination events in the hybrid progeny of 11 independent barley plants carrying the above target sequence after crossing with plants carrying a phiC31 expression cassette. Some of the hybrids displayed fully executed recombination. Excision of the *GFP* gene fostered activation of the *gusA* gene, as visualized in tissue of hybrid plants by histochemical staining. The recombinant loci were detected in progeny of selfed F_1_, even in individuals lacking the phiC31 transgene, which provides evidence of stability and generative transmission of the recombination events. In several plants that displayed incomplete recombination, extrachromosomal excision circles were identified. Besides the technical advance achieved in this study, the generated phiC31 integrase-expressing barley plants provide foundational stock material for use in future approaches to barley genetic improvement, such as the production of marker-free transgenic plants or switching transgene activity.

## Introduction

Plant genomic engineering took a big step forward after the introduction of site-specific recombinases, a group of enzymes that are capable of catalyzing reactions between two short, specific recombination sites [1], [2]. A specific characteristic of site-specific recombinases is that the outcome of the reaction depends on the placement of the recombination sites and their relative orientation [3]. Recombination between directly repeated target recognition sites results in a loss of the intervening DNA [4]. This technique has been used in plant systems to remove unwanted selectable marker genes [5], [6], resolve complex integration patterns [7], [8], [9], and activate genes by excising sequences that block the reading frame [10], [11], [12]. If the recognition sites are inverted, the recombination causes the sequence located in between to flip, which can be used to reconstitute a reading frame and thereby activate a plant transgene [13], [14]. Site-specific recombination occurring between recognition sites *in trans* can result in a reciprocal translocation of two linear DNA molecules or in a targeted integration if at least one DNA molecule is circular [15], [16].

The temporal or spatial control of recombination is enabled by the delivery of recombinases *in trans* through genetic crosses (hybridization) and removal of the recombinase in subsequent generations through segregation, a second round of transformation (either transient or stable) or transcriptional activation of the recombinase using inducible promoters [1], [2].

In general, all site-specific recombinases fall into one of two fundamental classes based on their evolutionary and mechanistic relatedness [2]. According to the active amino acid within the catalytic domain, these enzymes are known as tyrosine recombinases (or the “lambda integrase family”) or serine recombinases (or the “resolvase/invertase family”). Tyrosine recombinases cleave one strand of each of the two DNA molecules involved in the reaction and then exchange the strands, with the formation of a Holliday junction as a recombination intermediate [3]. Well-studied tyrosine recombinase systems include the bacteriophage Cre-*lox* and the FLP-*FRT* system from the 2-µm plasmid of *Saccharomyces cerevisiae*. Both have a long, proven track-record in different plant species, such as *Nicotiana tabacum*, *Arabidopsis thaliana*, tomato, maize, rice, wheat and turfgrass [2]. For biotechnological applications, it is important that tyrosine recombinases guide recombination between two identical recognition sites that remain unaltered after the reaction and thus persist as a substrate for the recombinase. As a result, the reaction is rendered fully reversible, although intra-molecular recombination (excision) is highly favored over inter-molecular reactions (integration) [17].

The serine recombinases catalyze a concerted process in which all four DNA strands are cut before being exchanged between the recombination sites and rejoined in the recombinant configuration [18], [19]. Serine recombinases recognize dissimilar recombination sites, commonly designated as *attB* (attachment site bacteria) and *attP* (attachment site phage). Because the recombination product is a hybrid sequence, known as *attL* or *attR*, that cannot serve as a target site for recombination, serine recombinases catalyze irreversible recombination in eukaryotic systems in which no accessory proteins are present [20]. Examples of serine recombinases used for manipulating plant genomes include the Gin recombinase of phage Mu in tomato [21], the β-six recombinase from *Streptococcus pyogenes* in *A. thaliana* and *N. tabacum* [22], the Bxb1 recombinase from mycobacteriophage Bxb1 in *N. tabacum* [23] and *A. thaliana* [24], the CinHRS2 system in *N. tabacum* [25] and the phiC31 integrase from the broad host range *Streptomyces* temperate phage. phiC31 was used in *Schizosaccharomyces pombe* [26] and several experimental animal systems, including *Xenopus laevis* [27], [28] and *Drosophila* [29], [30], and has become a key tool for gene therapy and other chromosomal engineering strategies in mammalian cells [31], [32], [33]. Compared with the well-established Cre-*lox* and FLP-*FRT* systems, however, the application of phiC31 in plants has been modest. The phiC31-*att* system has been applied to both integration and excision in the *N. tabacum* plastid genome [6], [34], [35] and to the excision of DNA fragments from *Arabidopsis* [36], [37]. More recently, phiC31-mediated excision of transgenes from the wheat genome was demonstrated [38].

In this article, we describe the use of the *Streptomyces* phiC31 integrase for the production of inheritable site-specific excision events in barley (*Hordeum vulgare*). To our knowledge, this is the first report of a heterologous site*-*specific DNA recombination system for genome manipulation in this crop species. Since barley is an important commercial cereal and a widely adopted experimental model for the temperate cereals [39], [40], we anticipate that the irreversible phiC31 system described here will be broadly applicable in future genome manipulation approaches in this species.

## Materials and Methods

### Vector Design

**Figure 1:**
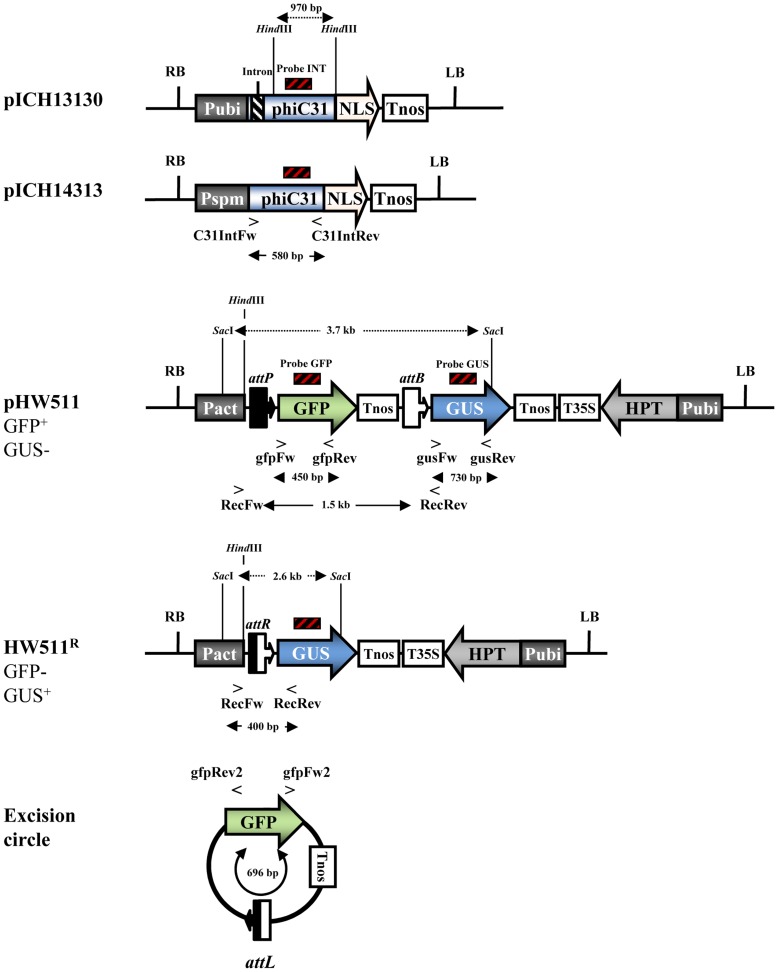
Genetic structure of the expression vectors and the recombinant locus. Note that only the T-DNA part of the vectors is illustrated (not drawn to scale). The integrase is expressed from the pICH13130 or pICH14313 constructs. The pHW511 vector harbors the *attP* and *attB* sequences, which serve as targets for recombination that results in the derivative locus HW511^R^ and the released excision circle. To select transgenic plants carrying the locus ICH13130 or ICH14313, co-transformation with the vector carrying an HPT selection marker was performed (p6U, not shown). Abbreviations: *phiC31*, phage phiC31 recombinase coding sequence [41]; *Pubi*, maize *ubiquitin 1* promoter; *Pspm*, promoter of the maize suppressor-mutator transposable element *spm*; *intron*, sequence derived from an intron of the *Petunia hybrida Psk7* gene (GenBank accession number AJ224165); *NLS*, SV40 T antigen nuclear localization signal, amino acids PKKKRKV [31]; *Tnos*, *nopaline synthase* terminator; *T35S*, cauliflower mosaic virus (CaMV) *35S* terminator; *GFP*, coding sequence for the green fluorescent protein; *GUS*, *β-glucuronidase* (*gusA*) gene; *Pact*, rice *actin 1* promoter; *attP* and *attB*, *Streptomyces* phage phiC31 recombination sites; *attR* and *attL*, hybrid products that originate from the recombination between *attP* and *attB; HPT*, *hygromycin phosphotransferase* gene.

The construction of the pBIN19-based vectors pICH14313 and pICH13130 (Figure 1) used to express the *Streptomyces* phiC31 integrase [41] has been previously described [14].

To construct the target vector pHW511 (Figure 1), the rice actin 1 promoter was amplified by Polymerase chain reaction (PCR) using the primers *F1actin* and *R1actin*, which incorporate unique *Stu*I and *Xma*I restriction sites (Supplemental Figure 1). The rice actin 1 promoter fragment was cloned into the pGus-AM plasmid (DNA Cloning Service, Hamburg, Germany) as the control element for a β-glucuronidase (*gusA*) coding sequence (including a StLS1 intron). The resulting construct was designated as pACTIN:GUS. The coding sequence, which was fused to the *nos* terminator and flanked by *attP* and *attB* sites in the direct orientation (*attP*:*GFP*:*nos*:*attB*), was synthesized by PCR, whereby the specific integrase target sites were designed as part of the primers *F1attPgfp* and *R1attBTnos*. The 1.1-kb PCR product was inserted in the pACTIN:GUS vector using *Hin*dIII and *Aat*II restriction sites, resulting in the sequence *attP*:*PActin:GFP:Tnos:attB:GUS:Tnos*. This 5-kb fragment is flanked by two *Sfi*I recognition sites, which were used for its directed ligation with the respective fragment of the binary p6U vector (DNA Cloning Service). p6U contains the hygromycin phosphotransferase (HPT II) gene as the selectable marker for plant transformation, which is controlled by the maize ubiquitin 1 promoter and a CaMV35S terminator. All constructs described in this paper were verified by DNA sequencing.

The plasmids were propagated in *E. coli* DH5α using standard DNA cloning methods [42]. For plant transformation, the vectors were precipitated on gold particles for biolistic delivery [14] or were transformed into the *Agrobacterium tumefaciens* strain AGL1 [43].

### Transgenic Barley Plants

Wild-type diploid barley (*H. vulgare,* cv. ‘Golden Promise’) plants were grown under controlled greenhouse conditions with 12 hours of light at 14°C and 12 hours of darkness at 12°C with a humidity of 80%. After 10–12 weeks of development, the plants were transferred to a greenhouse with at least 16 hours of light at 18°C and a corresponding period of darkness at 16°C and grown to maturity.

The parental plants carrying pHW511-, pICH13130- or pICH14313-derived loci, which were used for hybridization, were generated *via* inoculation of immature barley embryos with *A. tumefaciens* as previously described [44]. Transgenic calli were selected on callus induction medium containing 50 mg/l hygromycin B (Roche, Mannheim, Germany), and the transgenic plants were selected on regeneration medium containing 25 mg/l hygromycin B. In the case of the integrase vectors pICH13130 and pICH14313, co-transformation with the p6U plasmid was performed to allow selection on hygromycin.

For synchronous co-transformation of the target vectors and the integrase vectors, cultures of *Agrobacterium* containing pICH13130 or pICH14313 were mixed with cultures containing pHW511 immediately prior to the inoculation of immature barley embryos. Alternatively, co-transformation of barley with target and integrase vectors was performed *via* biolistic bombardment of immature embryos using the Biolistic PDS-1000/He Particle Delivery System (Bio-Rad, Munich, Germany) following a recently published protocol [14].

### Integrase Activity Assays

A virus-based GFP expression vector that is incapable of replicating (pICH16710; [14]) was delivered by biolistic bombardment into barley harboring an integrase transgene. The level of active integrase protein can be monitored by the frequency of integrase-mediated activation of the viral vector and the resulting GFP expression.

### Molecular Analysis of the Transgenic Plants

For total DNA isolation [45], leaf segments were harvested, frozen in liquid nitrogen and stored at −80°C. Homogenization was performed using a TissueLyser™ from Qiagen (Hilden, Germany).

PCR was performed in a thermocycler (DNA-Engine™ PTC-0200, Bio-Rad, Munich, Germany), involving an initial denaturing step at 95°C for 5 minutes followed by 35 cycles (94°C for 30 s; 60°C for 30 s; 72°C for 1–2 min). The amplified fragments were run on 1–1.5% agarose gel containing 4 µg/100 ml ethidium bromide.

The positions of the primer binding sites are depicted in Figure 1. The primer sequences are given in Supplemental Figure 1. The primers were used as follows: i) to detect the *gusA* gene and production of the GUS probe for DNA gel blots: *gusFw* and *gusRev*; ii) to detect the *GFP* gene and production of the GFP probe for DNA gel blots: *gfpFw* and *gfpRev*; and iii) to show the presence of the integrase-encoding sequences of pICH14313 and pICH13130 and production of the INT probe for DNA gel blot: *C31IntFw* and *C31IntRev*.

The site-specific recombination was molecularly confirmed by PCR in which the excision footprint sequences, including the hybrid recombination product *attR*, were amplified with the *RecFw* and *RecRev* primers. In the case of the non-recombinant locus HW511, the PCR amplification resulted in a 1.5-kb fragment (Figure 1). If recombination occurred at the target *attP* and *attB* sites, the derivative locus HW511^R^ was produced, and a 400-bp fragment was obtained. Detection of an excision circle was achieved using the outwards primers *gfpFw2* and *gfpRev2*. An amplification product of 696 bp can only be synthesized in the case of a circular fragment, *attL-GFP-Tnos* (Figure 1).

For sequence analysis, the PCR products were subjected to direct DNA sequencing by GATC Biotech (Konstanz, Germany).

The DNA gel blots were conducted according to standard protocols [46]. The primers used to produce the probes are given in Supplemental Figure 1. The DNA fragments were separated using 0.6% agarose gels and transferred onto a nylon membrane (Biodyne B; Pall, USA). After blotting, the membranes were hybridized with [32P]-labeled DNA fragments. To estimate the copy number of the target locus HW511, total DNA was digested with *Hin*dIII and hybridized with either the GFP or GUS probe. These strategies resulted in fragments containing the homologous vector sequence along with a genomic DNA stretch of unpredictable length, which is expected to be different for every individually integrated vector sequence. These size differences allow the transgene copy numbers to be assessed. The presence of pICH13130- or pICH14313-T-DNA was also confirmed by digesting total barley DNA with *Hin*dIII, which releases a 970-bp fragment containing the integrase sequence covered by the INT probe.

To detect the recombinant locus HW511^R^, total plant DNA was digested with *Sac*I. In the case of HW511^R^, a 2.6-kb fragment covered by the GUS probe is released, whereas the unaltered target locus results in a fragment of 3.7 kb homologous to GUS and GFP.

### Detection of Reporter Gene Expression

The transgenic plants were analyzed for GUS activity using histochemical β-glucuronidase staining [47]. Leaves, flower organs, calli and embryos were incubated overnight in 96-microwell plates at 37°C with phosphate buffer (50 mM sodium phosphate pH 7.0, 1 mM EDTA, 0.1% Triton-X-100) containing 1 mM 5-bromo-4-chloro-3-indolyl-β-D-glucuronic acid (X-Gluc). The chlorophyll was removed from the leaf material by repetitive treatment in 96% ethanol for 2 hours at 60°C.

Tissues expressing GFP were viewed under UV illumination generated by a Leica DM IL microscope with filter sets for GFP plant fluorescence (excitation filter, 470_40 nm; barrier filter, 525_50 nm).

## Results

### Vector Design for *in planta* Detection of Integrase Activity

To test the functionality of the phiC31-*att* recombinase system in barley plants, the strategy illustrated in Figure 1 was employed. Hereafter, the plasmids will be designated as pHW511, pICH13130 and pICH14313. If not explicitly stated otherwise, the term “integrase vector” refers to both pICH13130 and pICH14313. The corresponding chromosomal loci of the transgenic barley plants are designated as HW511 (for the non-recombinant locus), HW511^R^ (for the recombinant locus) and ICH13130 or ICH14313 for the integrase source.

The pHW511 construct contains a GFP transgene, which is controlled by the constitutive actin 1 promoter from rice and a nos terminator (Figure 1). A promoter-less *gusA* gene is located downstream. Transgenic plants harboring HW511 are expected to produce the GFP protein but no *gusA* gene product. The target insert *GFP*-*Tnos* is flanked by *att* recognition sites for the *Streptomyces* phage phiC31 integrase, which itself is expressed from a second locus ICH13130 or ICH14313. If such a locus is present in the same cell, an active integrase may be produced in the cytoplasm and be subsequently imported into the nucleus due to its nuclear localization signal, where it will possibly catalyze an irreversible site-specific recombination event between an *attP* and *attB* site at the target. As a result, the *GFP*-*Tnos* fragment will be excised from the chromosomal locus HW511, and the *gusA* gene will be fused to the rice actin 1 promoter and hence activated. Because the recombination products *attR* and *attL* are not substrates for the phiC31 integrase, the reaction is irreversible.

It should be emphasized that Figure 1 illustrates an “idealized” genetic transformation event in which one copy of an intact target locus is distinctly integrated into the genome.

### *In vivo* Evaluation of the phiC31-*att* System

With the goal of verifying the potential functionality of the integrase and target vectors in barley, we delivered the plasmids jointly into barley tissue. This was achieved either by co-bombardment of embryos using a mix of both the integrase and the target plasmids or by co-cultivation of immature embryos with mixed *Agrobacterium* cultures.

As a result, GUS expression was found in co-bombarded embryos and in the majority of the embryo-derived calli that developed after co-transformation, but not in control experiments where only pHW511 was used (Supplemental Figure 2). PCR conducted with the primers *RecFw* and *RecRev* with DNA prepared from embryos or callus that displayed GUS expression resulted in a fragment of 400 bp, thus indicating a recombination event (data not shown). From our results we concluded that the vectors are functional with regard to the marker genes and the recombination system and are suitable for stable transformation and hybridization experiments.

**Figure 2:**
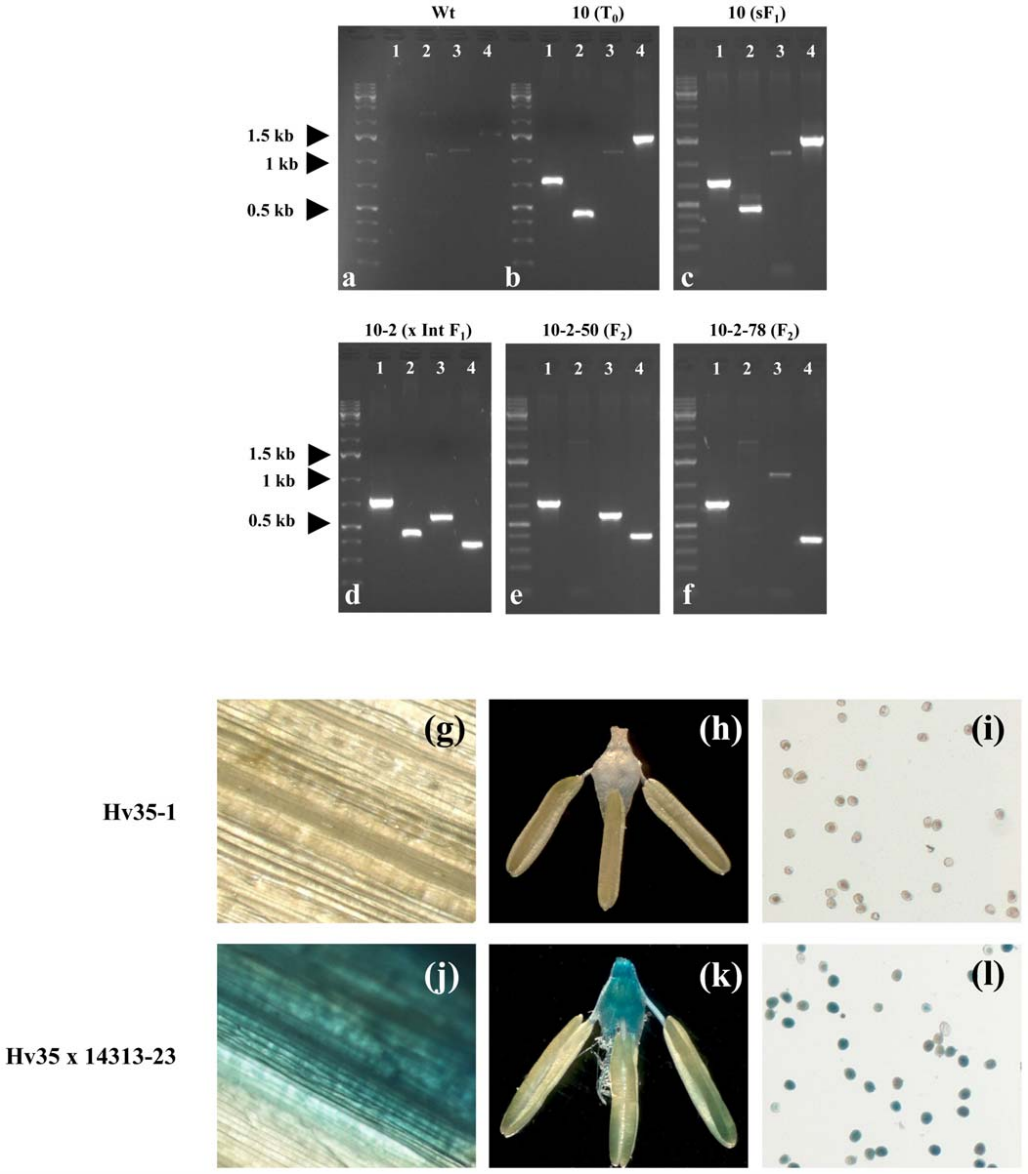
Molecular and phenotypic analysis of hybrids obtained by combining transgenic plants carrying target and integrase loci. (a–f) PCR analysis was performed using the *gusFw* and *gusRev* (lane 1), *gfpFw* and *gfpRev* (lane 2), *C31IntFw* and *C31IntRev* (lane 3), and *RecFw* and *RecRev* (lanes 4) primers on total DNA from untransformed plants (a; *Wt*) and total DNA from Hv10 and its descendants (b–f); (b) primary transformant (*T_0_)* containing the HW511 locus; (c) *sF_1_*, plant obtained by selfing of Hv10 containing the HW511 locus; (d) hybrid F_1_ carrying a phiC31 integrase and a recombined locus HW511^R^; (e) F_2_ plant that inherited the recombined locus HW511^R^ and the integrase locus ICH14313; (f) F_2_ plant that inherited the recombined locus but no integrase locus. The positions of the primer binding sites are given in Figure 1. (g–l) Analysis of GUS expression in primary transgenic plants (T_0_) carrying the HW511 locus and no integrase (g, h, i) and a hybrid F_1_ plant (Hv35×14313–23) that harbors a recombined locus (j, k, l) using leaf tissue (g, j), ovary and stamen (h, k) as well as pollen (i, l).

### Development of Parental Plants for Hybridization

A total of 42 transgenic barley plants harboring the target locus HW511 were obtained *via Agrobacterium-*mediated transformation. DNA gel blot analysis was used to estimate the transgene DNA copy number. Furthermore, the plants were assayed for the presence of an active HW511 locus by fluorometric GFP assays (data not shown). Plants that showed high GFP activity were selected as parents for hybrid crosses, with preference being given to plants with a low transgene copy number.

As sources for the integrase, 20 transgenic barley plants harboring the integrase locus pICH13130 and 6 plants carrying the integrase locus ICH14313 were produced. All transformants were evaluated for the presence of an active integrase by a previously published transient viral-based assay [14] (see Materials and Methods; examples are shown in Supplemental Figure 3). The plants that displayed the highest activity of recombinant phiC31 integrase were selected as pollen donors for sexual hybridization with plants carrying the target vector pHW511.

**Figure 3:**
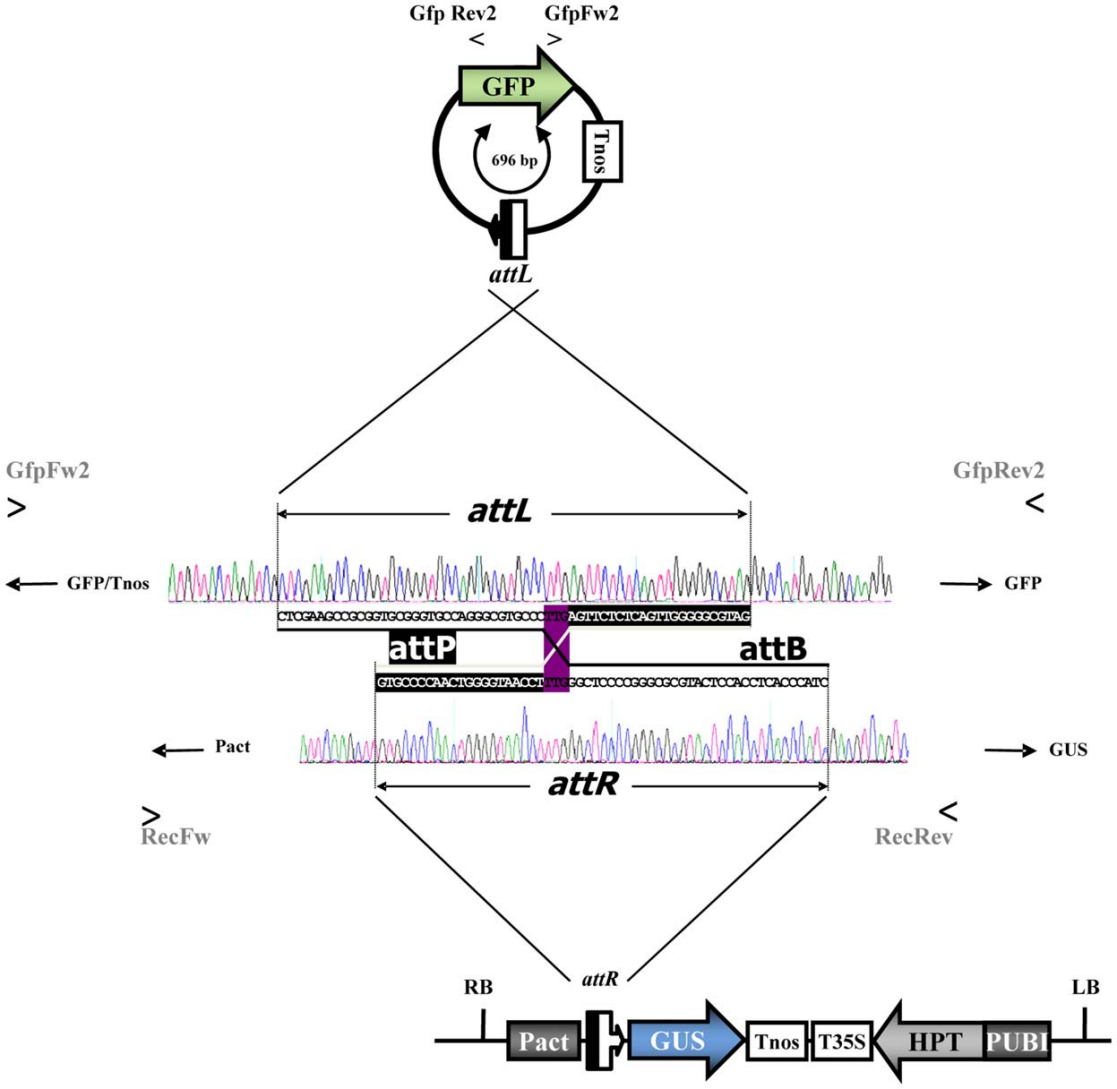
Footprint-sequence analysis of HW511R and the excision circle. Footprint sequences of hybrid *attR* or *attL* resulting from a reaction between *attB* (black) and *attP* (white). Both recombination products share an identical 3-bp long central core, ‘TTG’, where the crossover occurs. Electropherograms were taken from sequencing analysis. PCR-fragments were sequenced by using the primers *GfpFw2* and *GfpRev2* or *RecFw* and *RecRev*. The positions of the primers and the adjacent vector parts are indicated.

None of the barley plants expressing the phiC31 integrase exhibited phenotypic differences compared with non-transgenic counterparts grown under the same conditions. This indicates that expression of the phiC31 recombinase does not entail apparent disadvantage in terms of plant development. Plants were investigated over three generations (T_0_–F_2_).

### phiC31-mediated Transgene Excision in Hybrid Progeny

For the production of co-transgenic hybrids, T_0_ plants carrying the target sequence were used as pollen acceptors for crosses with the integrase sources. In total, 14 independent plants carrying the target locus HW511 were crossed using four different plants harboring an integrase expression cassette. Crosses of T_0_ plants that carried a single copy of the integrase with T_0_ plants that carried a single copy of the target vector resulted in F_1_ plants of which approximately 25% were co-transgenic. We identified 80 co-transgenic hybrid F_1_ plants (derived from 14 independent T_0_ target plants) that carried both the target locus and integrase locus (summarized in Table 1). Hybrid F_1_ plants were screened for site-specific excision events *via* PCR. The design of the primers allows for the production of PCR fragments of specific sizes only in the presence of the derivative locus HW511^R^ (Figure 1). The results of several PCR experiments are exemplified in Figure 2. Plants were examined at an age of 2–4 weeks or alternatively by analyzing flag leaf samples of three different generative tillers of three-month-old plants.

**Table 1:** Analysis of hybrid F_1_ plants co-transgenic for both integrase and target sequence.

Cross of T_0_ plants (HW511×Integrase)	Copy No. of target vector	Hybrid F_1_ plants analyzed	+ Recombination* ^2^	− Recombination* ^3^
*Single-copy target* * ***^1^***				
Hv6×14313–23	1	6	0	6
Hv10×14313–23	1	4	1 (1)	3
Hv18×14313–24	1	3	3 (2)	0
Hv28×14313–23	1	14	4 (3)	10
Hv35×14313–23	1	3	1 (0)	2
Hv36×14313–24	1	9	3 (1)	6
Hv37×14313–24	1	3	1 (0)	2
*Multiple-copy targets*				
Hv40×14313–14	3	6	0	6
Hv3×14313–23	4	3	2 (1)	1
Hv9×13130–5	4	5	0	5
Hv30×14313–23	4	10	5 (3)	5
Hv12×14313–23	>4	2	1 (1)	1
Hv13×14313–23	>4	8	7 (7)	1
Hv15×13130–5	>4	4	2 (2)	2

The results of the PCR analyses are summarized. The copy number of the target sequence was estimated by DNA gel blot analysis.

Independent co-transgenic hybrid plants carrying both the HW511 and either the ICH13130 or the ICH14313 loci, or a recombinant derivate were included in the analysis. Descendants that lost the target locus or the integrase locus or both due to segregation in meiosis of T_0_ plants are not listed in the table.

“+”/“−“ indicates the presence/absence of recombination.

* **^1^** Primary transformants (T_0_) that were hemizygous for the target locus were crossed with the integrase lines.

* **^2^** PCR using primers *RecFw* and *RecRev* resulted in the production of a 400 bp fragment containing *attR* (Fig. 1 and 2). The number of individuals that contained a recombination event and a non-recombinant locus (which was identified by PCR-amplification of GFP with the primers *gfpFw and gfpRev* and/or by the amplification of a 1.5 kb fragment using primers *RecFw* and *RecRev*) is indicated in *brackets.*

* **^3^** PCR using primers *RecFw* and *RecRev* resulted in the production of a 1.5 kb fragment containing *GFP* (Fig. 1 and 2).

Among the 80 hybrid plants, we identified 30 individuals (37%; derived from 11 independent T_0_ target plants) that carry a recombinant locus (Table 1). From these findings, we concluded that, in barley, a phiC31 integrase expressed from the chromosomal loci ICH13130 or ICH14313 can foster the excision of the target insert *in trans*.

In the majority (21 of 30) of the plants displaying recombination events, an additional non-recombinant locus (HW511) was identified by the detection of a *GFP* sequence. Furthermore, in several cases, the derivative locus HW511^R^ was detected only in some parts of the plants. We assume that both observations are most likely attributed to some of the F_1_ plants being genetic chimeras that contain both recombinant and non-recombinant tissues. The remaining plants harbored only a recombinant locus, which is most likely caused by an early excision event, possibly in the zygote. However, analysis of hybrid F_1_ plants is generally hampered because only a part of the plant tissue can be analyzed without sacrificing the respective individual, thereby leading to the possibility that a chromosomal locus might not be identified if a sector of the plant is not included in the analysis.

### Activation of *gusA* in the Hybrid Progeny

Tissues from all plants carrying a recombinant locus were subjected to β-glucuronidase (GUS) staining (Figure 2 g–l). In the vast majority (>90%) of plants with recombinant loci, an active *gusA* gene was detected (3 j–l), whereas the control plants always failed to produce the enzyme (3 g–i). From these data, we deduce that the decryption of *gusA* is accomplished by removal of the excision target. Histochemical analysis of barley leaf tissue is known to be cumbersome due to low penetration of the substrate. Thus, we conclude that the blue sectors that appeared in some of the leaves after staining were caused by uneven distribution of the substrate rather than by chimerism with regard to the recombinant locus. GUS expression was also verified in ovaries (Figure 2 k) and pollen (l). Expectedly, only a portion of the pollen displayed a recombinant phenotype due to segregation of the recombinant locus HW511^R^ during meiosis. Plants harboring a single recombinant target locus displayed approximately 50% of pollen with recombinant phenotype (Hv35, Fig. 2l). However, de-staining of barley pollen was difficult and not complete in some cases.

### Excision Footprint Sequencing Analysis

The 400-bp *RecFw*/*RecRev* amplification products were subjected to DNA sequencing. All 11 recombinant plants that resulted from hybridization were included in this examination. The footprint of the excision always perfectly matched the predicted sequence of the recombination event between two *att* recombination sites, including the *attR* sequence, which is adjacent to the newly linked sequence of the chromosomal integration locus (Figure 3). From these results, we infer, first, that the polymorphisms revealed in the length of the PCR fragments are a reliable indicator of phiC31 integrase-catalyzed site-specific recombination at the HW511 locus and, second, that phiC31-mediated recombination is an accurate process that leads to a predictable excision footprint in the barley genome.

### The Target Locus HW511 does not Spontaneously Recombine in the Absence of the Integrase-expressing Locus

To assay the stability of the HW511 locus, 65 plants resulting from backcrosses and 156 plants resulting from self-pollination were examined by PCR analysis and phenotypic assays, including descendants of the 11 T_0_ target plants that were used for the above hybridization with the integrase sources. There were no indications of any “spontaneous” (e.g., recombinase-independent) rearrangement of the target locus HW511. To conclude, the “molecular safety lock” that is created by the *GFP-Tnos* “block” appears to permit a tight encryption of the *gusA* gene, and GUS expression in F_1_ hybrids results solely from the integrase-mediated removal of this sequence.

### Generative Transmission of the Recombinant Locus

To investigate whether the recombination events are sexually transmitted, we examined 196 F_2_ descendants obtained through selfing of plants belonging to the 11 recombinant hybrid F_1_ families. In seven F_2_ families, the recombinant locus HW511^R^ was found by PCR analysis (Table 2). We identified 139 F_2_ plants (70%) carrying a recombinant locus. Its absence, however, was expected for some F_2_ plants as a result of segregation because the F_1_ generation is hemizygous for the target loci. The occurrence of F_2_ plants displaying both recombinant and non-recombinant loci (Class I and II, respectively) implies that phiC31-mediated recombination has occurred but that not all target inserts have been excised. Most expectedly however, recombination events that occur late in the previous generation can result in the formation of both non-recombined and recombined gametes, transmitting both loci to the subsequent generation. Furthermore, in the continued presence of the phiC31 integrase, the loci might recombine in different generations, thereby leading to genetic chimeras. In the case of F_1_ plants carrying multiple loci (Hv12, Hv13 or Hv15), the presence of both recombinant and non-recombinant loci might reflect the inability of the phiC31 integrase to excise certain targets, possibly as a consequence of a different accessibility of the target loci due to different chromosomal locations (“position effects”). Alternatively, or additionally, the GFP gene may be retained as the final product of a recombination event in which a complex T-DNA locus is converted into a less complex locus. Indeed, DNA gel blot analyses strengthen this assumption (Supplemental Figure 4).

**Figure 4:**
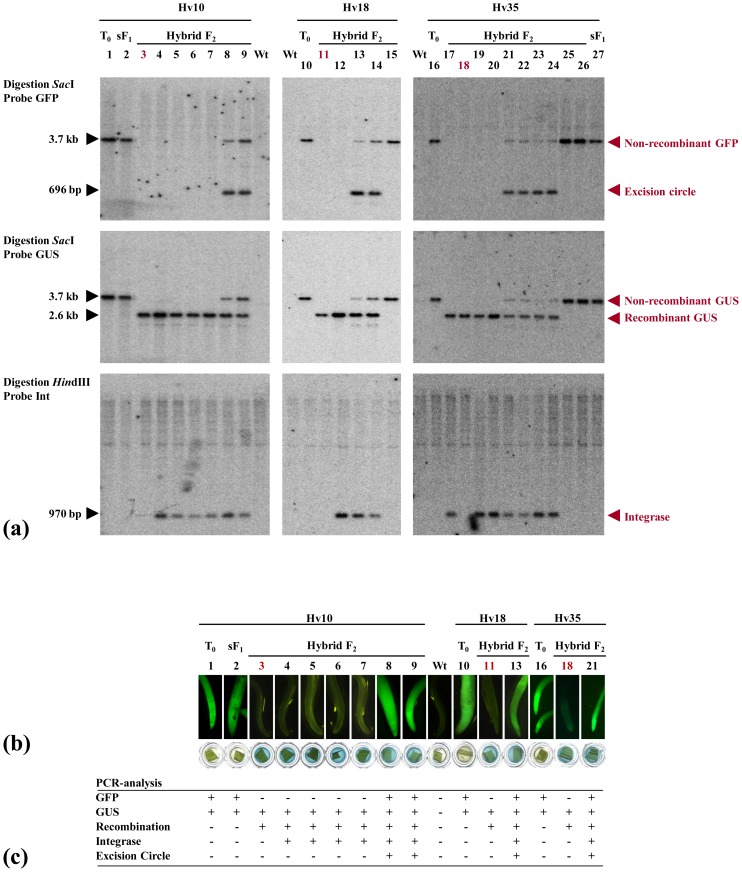
Molecular and phenotypic analysis of hybrid F_2_ plants. (a) DNA gel blot analysis of three transgenic plants containing a single copy of the target locus (*Hv10, Hv18, Hv35*) and their descendants obtained by selfing *(sF_1_*) or crossing with transgenic plants carrying an integrase locus (*hybrid F_2_*). Plants that contain a recombined locus and no integrase are highlighted. For DNA preparation, 5-week-old plants were used. The designated restriction enzymes and sequence regions homologous to the hybridization probes are depicted in Figure 1. As controls, untransformed plants (*Wt*) are included. (b) Reporter gene assays. To monitor GFP expression, fluorescence microscopy was performed using root tips. β-glucuronidase (GUS) staining was carried out using leaf material. (c) PCR analysis.

**Table 2:** Sexual transmission of recombinant loci.

Target sequence carrying parent	No.of hybridF_2_ plants analyzed	Class I + Integrase+ Recombination+ GFP	Class II − Integrase+ Recombination+ GFP	Class III + Integrase+ Recombination− GFP	Class IV − Integrase+ Recombination− GFP	Class V− Recombination
Hv10	68	34	11	4	1	18
Hv18	41	28	2	1	1	9
Hv28	7	5	0	0	0	2
Hv35	47	24	0	3	1	19
Hv12	10	8	0	0	0	2
Hv13	12	6	0	0	0	6
Hv15	11	7	3	0	0	1

“+”/“−” indicates the presence/absence of a recombinant locus, a GFP transgene or integrase transgene (assayed by PCR and DNA gel blot analysis).

Notably, 19 F_2_ plants carrying a recombinant locus were lacking the integrase due to independent segregation (Class II and IV). In these cases, it can be excluded that the phiC31-mediated site-specific recombination events did emerge *de novo* in the F_2_ plants, which provides compelling evidence of sexual transmission of those events. Therefore, we deduce that the phiC31 system is suitable for creating integrase-free barley plants that harbor only the recombinant locus.

### Analysis of Recombination Events in the F_2_ Generation

DNA gel blot analysis was used to examine progeny from the Hv10, Hv18 and Hv35 F_1_ plants (Figure 4 a). In agreement with the PCR analyses (b), in a number of plants, the excision target *attP-GFP-Tnos-attB* was completely eliminated (lanes 3–7, 11, 12, and 17–20). Three of these plants lost the phiC31 transgene through segregation (lane 3, 11, 18), which indicates germinal transmission of the excision event. HW511^R^ is represented by a 2.6-kb fragment homologous to the GUS probe. All plants carrying such a locus contain an active *gusA* gene, as demonstrated by histochemical staining with X-Gluc (Figure 4 c). Conversely, the T_0_ control plants (lanes 1, 10, and 16) displayed only the unaltered targeting sequence (3.7-kb fragment homologous to GUS/GFP) and no β-glucuronidase expression. Some F_2_ plants inherited only the non-recombinant target locus (lanes 15; 25–26). Moreover, several F_2_ individuals were chimeric for recombinant and non-recombinant loci, as indicated by the presence of both the 2.6-kb GUS and 3.7-kb GUS/GFP fragments.

### Plants with Incomplete Recombination Contain a Stable *attL-GFP-Tnos* Excision Product

In the DNA gel blot analyses of the plants displaying a chimeric pattern, a prominent GFP fragment of approximately 0.7 kb was detected (Figure 4, lanes 8, 9; 13, 14; 21–24). The presence of this fragment was associated with the presence of both the integrase and the undeleted locus. Moreover, the autoradiogram showed that, in some of these plants, there was a certain negative correlation between the intensity of the 0.7-kb signal and that of the “non-recombinant” 3.7-kb GFP/GUS signal (lanes 13, 14 and 21–24). Therefore, we speculated that the 0.7-kb signal represents the DNA fragment that was excised from the chromosome by the phiC31 integrase. The assumption was strengthened by the observation that DNA of corresponding size could be also detected when undigested DNA was blotted and hybridized with the GFP probe (Supplemental Figure 5). To prove our hypothesis, PCR was carried out on total barley DNA using the outward primers *GFP Fw2* and *GFP Rev2* (Figure 1). DNA from all plants carrying the 0.7-kb fragment gave rise to a PCR fragment of 696 bp, thus demonstrating the circular nature of the template. The amplification products were sequenced, and the results were consistent with the deduction of a circular *attL-GFP-nos* excision product (Figure 3). As a control and to rule out the possibility of contamination, the experiments were conducted with total DNA from non-transformed plants, T_0_ plants (Figure 4, lanes 1, 10, 16), progeny from selfed T_0_ plants (lane 2 and 27) and F_2_ plants showing complete removal of the GFP gene (lanes 3–7; 11–12 and 17–20) and F_2_ plants that carry no integrase (lane 15; 25–26). In all of these controls, PCR using the primers *GFP Fw2* and *GFP Rev2* failed to amplify a DNA fragment.

## Discussion

In this study, we describe the successful use of the phiC31 integrase system for excising transgenes from the barley genome through site-specific recombination. Barley is the fourth most important cereal worldwide and has a significant agro-economic impact, with a harvested area of >47 million hectares (FAOSTAT, 2012; http://www.fao.org/faostat). In addition, barley has a long-standing history as an experimental model system, representing a number of small grain cereal species. In recent years, a substantial body of genetic and genomic resources has been generated and collected worldwide [39], [40]. The barley genome exhibits high collinearity with other *Triticeae* species (e.g., wheat, rye, and ryegrass), with which it also shares numerous agronomic traits. However, its diploid character makes barley considerably more amenable to examinations of classical and molecular genetics. Furthermore, the development of efficient transformation protocols has stimulated the establishment of numerous efforts for functional gene analysis, for engineering transgenic barley with improved crop quality or for molecular farming [48], [49], [50], [51]. For advanced transgenic technologies, particularly in the context of increased control over transgene expression in crops, tools that facilitate the excision and integration of transgenes with a strictly “guarded” directionality would be beneficial. Thus, we consider the development of irreversible prokaryotic site-specific recombination systems for barley to be an imperative goal. The phiC31 system lacks a readily reversible reaction in non-bacterial systems. Integrases control the reversibility of the reaction by recombining dissimilar target sites. In contrast, the well established Cre system has necessitated modifications to the lox target sites to reduce or obviate re-excision [52], [53], [54].

The results reported in this study suggest that the expression of phiC31 integrase, when expressed in transgenic barley plants, fosters the excision of a transgene of a target sequence that resides on another chromosomal locus in the same cell. As a prerequisite for practical applications, the recombinant loci are faithfully inherited by subsequent generations. Furthermore, our results accomplish the concept of an induced ”genetic switch” (GFP→GUS) that is triggered by the action of a recombinase, which itself can be removed, preferably through generative segregation, as soon as it is no longer needed.

There have been reports of undesirable effects associated with the expression of the site-specific recombinase Cre in several species belonging to the *Solanaceae* family [55], [56], [57], [58]. Such phenotypic effects can be due to the presence of the Cre protein itself or to its activity on spurious cryptic *lox* or *lox*-like sites in the plant genome. Cryptic pseudo *attP* sites have been speculated to be involved in integrase-mediated chromosomal rearrangements and integrations, similar to phenomena that have been commonly found in mammalian cells [59], [60]. Cryptic recognition sites in plants were identified through sequencing analysis [36]. However, several reports exist in which phenotypical abnormalities upon Cre expression have also been found to co-segregate with Cre gene in progeny and consequently, the phenotype is reversed upon segregation of Cre gene [55], [58]. In such cases it is likely that the phenotype does not emerge from chromosomal rearrangements but from the Cre expression itself. The barley plants that constitutively express the phiC31 integrase under control of either the maize ubiquitin promoter (DH13130) or the maize *spm* promoter (DH14313) did not show apparent phenotypic abnormalities (over three generations). These results are in accordance with data reported for the expression of both Cre and phiC31 in wheat [7], [38].

In the case of the co-transgenic hybrid F_1_ barley plants, 37% displayed a phiC31-mediated recombination event. This frequency is lower than that observed in a similar study of wheat, in which 96% of co-transgenic plants harbored a recombinant locus [38]. However, comparing these results is difficult because the transgenic wheat plants were produced by biolistic bombardment and carried considerably more target sequences for recombination than the barley plants produced by *Agrobacterium*-mediated transformation. F_2_ progeny resulted from selfed co-transgenic (recombinant) F_1_ hybrids were analyzed for the presence of recombinant loci, whereby the loss of transgenic loci is expected in a part of the progeny as a result of segregation alone (F_1_ is hemizygous for the transgenic locus). The proportion of barley F_2_ plants that displayed recombination was 70% in the present study and 65% in the case of wheat [38]. In an earlier study, pICH14313 was used to excise transgenes from the genome of *A. thaliana* [37]. The recombination frequency in co-transgenic F_1_ progeny was 42% and the proportion of F_2_ plants that displayed recombination was 64%. It should be noted that the target vectors used in the previous studies have a different structure than locus HW511, as they contained multiple recombination targets and varying distances between the *att* sites. With regard to germinal transmission efficiency, the F_2_ data has to be interpreted with care since it cannot be excluded that recombination may occur *de novo* in some F_2_ plants that still carry an integrase.

In a number of co-transgenic barley F_1_ hybrids produced in the present study (Hv6, Hv9, Hv40), no recombination was detected, and some of the recombinant plants failed to inherit the recombinant locus to the next generation. In addition, a number of plants containing complex loci displayed chimeric recombination patterns. Several mutually non-exclusive reasons might explain these varying results of recombination: (a) phiC31-mediated site-specific recombination events may occur in different cells and at various time points during plant development, (b) different recombination products may occur in the case of complex patterns, (c) the recombination efficiency may depend on the genomic position of the target sequences. For plants, it was postulated that condensed chromosomal DNA has a reduced accessibility for enzymes that are involved in recombination processes [61]. Similar results were published for animal systems [62]. Thus, it can be speculated that variations in the efficiency of site-specific recombination between different targeted loci might be due to different chromosomal positions. Such ‘position effect’ may be a result of DNA methylation in the *att* sites, hampering the binding of the phiC31 protein, or the *att* sites may become less approachable in certain locations of the host genome. It was speculated that such effects were responsible for the variability of recombining different *FRT* sites in the rice genome [63].

On the basis of our results, it appears that the phiC31 recombinase mediated excision in barley does not fully approach the efficiency of Cre-*lox* systems that was achieved in several studies conducted in other plant systems [64]. phiC31 mediated recombination occurs at a level that seems to be suitable for practical applications. However, it is difficult to give a precise quantitative assessment of the phiC31 activity in comparison to other recombinases since only a modest number of different target locations were analyzed and direct comparison to other systems (like Cre-*lox* or FLP-*FRT*) was not addressed. It might be possible that, in future work, through optimization (e.g. the use of alternative promoters or nuclear localization signals) phiC31 can be enhanced to higher levels of activity in barley and thus elevate the efficiency of the excision reaction.

Among the various applications for site-specific recombinases, the elimination of unwanted sequences from transgenic plants, most notably selectable marker genes, has gained special interest [5], [65]. To substantiate claims about the removal of the phiC31-mediated transgene, tracking the fate of the deleted DNA is essential. There is a debate about the persistence of the excised circular product from site-specific recombinases. Several reports describe the maintenance of excised DNA in non-dividing cells in animal systems [66], [67]. In plants, a Cre-mediated deletion product was maintained as an extrachromosomal circular molecule in rare cases in wheat [68]. An excision circle was also identified in somatic tomato cells [58], but the circles were unstable and were soon lost after conception. In another study [69], the FLP-FRT site-specific recombination system was used to excise and activate a previously integrated homing endonuclease in maize zygotes and/or developing embryos. An active endonuclease was expressed; nevertheless, the extrachromosomal DNA disappeared over time.

Other authors studying this aspect of recombination have found no indications that a released DNA fragments remain extrachromosomally [9].

Our data suggest that the observed phiC31 excision products are not subjected to an immediate cellular degradation after their emergence. We hypothesize that degradation of the excision product by non-specific nucleases is prevented, possibly due to the presence of a native chromatin structure. Such a process may depend on the nature of the DNA, the involved recombinase and the cellular environment and therefore might have differing efficiencies for various studies and plant species. In this study, excision circles were detected in descendants of three independent T_0_ plants, all of which displayed incomplete excision of the target sequence. F_2_ plants that inherited only a recombined locus from the previous generation (Figure 4, lane 3–7; 11, 12; 17–20) did not contain a deletion product. The same result was obtained for F_2_ plants that inherited a non-recombinant target locus but no integrase (Figure 4, lane 15; 25–26) and for F_1_ plants that contained only the recombined locus. Based on the summary of our data, we speculate that the excision circles were generated *de novo* in such F_2_ plants that contain both a non-recombinant target locus as a substrate for recombination and an integrase transgene that is capable of excising the circle from the target locus. We speculate that these recombination events most likely occurs in non-dividing somatic cells at a late stage of plant development, which could explain why a “dilution” of the excised DNA during mitosis did not take place and why a prominent signal appeared on the DNA gel blot autoradiogram. Thus far, to our knowledge, there have been no reports about the maintenance of deleted DNA in dividing cells. However, the persistence of excised products in somatic, non-dividing cells may pose biosafety concerns; in particular because in the case of reversible systems such as Cre-*lox*, the excised molecule can theoretically be propagated through cycles of reinsertion into its original genomic location. This possibility should be remote in the case of non-reversible systems such as phiC31 integrase. Still, further and more comprehensive studies should be performed to address these issues in more detail.

The objective of this study was to demonstrate the feasibility of using the phiC31 integrase recombination system to produce transgenic barley plants that carry a stable recombinant locus. Our data imply that the phiC31-*att* system is appropriate for accomplishing this goal. Our results not only represent the first implementation of gene excision and recombinase-induced gene switching in barley but may also constitute the basis for a variety of future advanced technologies for barley genome engineering. We assume that the availability of a variety of site-specific recombination systems will foster complex genomic engineering strategies for barley genome improvement. Thus, we consider phiC31 to be a valuable alternative/addition to the recombinase systems that are already established in plant systems, particularly in light of advanced technologies for GM plant production. These technologies include the use of multiple recombination systems and strategies for multi-gene stacking and deletion; some complex strategies have been suggested that incorporate both reversible and irreversible recombination systems [1], [2], [5], [70], [71]. We believe that the transgenic plants generated in the present study for expression of phiC31 integrase will provide a technical foundation for future research in the field.

## Supporting Information

Figure S1 **Names and sequences of oligonucleotide primers used in this study.**
(PDF)

Figure S2 **In vivo evaluation of vector constructs.** (a–f) Analysis of barley embryos bombarded with the pHW511 plasmid (a, b) or co-bombarded with pHW511 and either pICH14313 (c, d) or pICH13130 (e, f). After 24 hours, the first GFP signals were detectable (Figure 2 a, c, e). β-Glucuronidase (GUS) staining of the embryos was performed 2 days after bombardment and revealed GUS signals in embryonic tissue that had been co-bombarded with both plasmids, pHW511 and an integrase vector, thereby indicating recombination (2 d, f). In contrast, in the control experiments carried out with pHW511 only, no GUS signal was observed (2 b). (g–l) Analysis of callus tissue derived from embryos that were bombarded with pHW511 (g, h) or co-bombarded with pHW511 and either pICH14313 (i, j) or pICH13130 (k, l). GUS expression was found in the majority of the embryo-derived calli that developed after co-transformation (j, l), but not in the calli of the control experiments (h). GFP expression was, as expected, ubiquitously present (2 g, i, k). (a, c, e, g, i, k) display fluorescence microscopy images; (b, d, f, h, j, l) present results obtained by β-glucuronidase (GUS)-staining.
(PDF)

Figure S3 **Transient assays for integrase activity.** Bombardment of T_0_ plants harboring the integrase locus ICH13130 (a) or ICH14313 (b) with the viral vector pICH16710 which carries a GFP expression cassette and whose replication is activated after phiC31 integrase catalyzed recombination [14]. (c) Control experiment in which an untransformed plant was bombarded with pICH16710. (d, e) GUS staining was performed on leaf material of T_0_ plants harboring the integrase locus ICH13130 (d) or ICH14313 (e) after bombardment with the target vector pHW511.
(PDF)

Figure S4 **Resolution of complex loci through recombination.** (a) Total DNA of plants containing multiple copies of the target locus was digested with the restriction enzyme *Hin*dIII and hybridized with probes GUS, GFP, and INT. This strategy allows for detection of a constant integrase-fragment and estimating of the copy-number of the target locus. Different patterns of F_2_ progeny plants can be explained by segregation of unlinked recombinant or non-recombinant loci, different outcomes of the recombination in the case of complex integration patterns or, recombination events that occur late in the development of the F_1_ plant and are independently inherited to the individual progeny plants. For comparison, the analysis of primary transformed plants carrying a single-copy of the locus HW511 are documented (b).
(PDF)

Figure S5 **Detection of excision circles using undigested DNA.** DNA gel blot analysis was carried out using undigested total DNA of transgenic plants containing a single copy of the target locus *Hv35* and their descendants obtained by selfing *(sF_1_*) or crossing with transgenic plants carrying an integrase locus (*hybrid F_2_*). As controls, untransformed plants (*Wt*) are included. The membrane was hybridized with the probe GFP. The arrangement of plants is identical to that in Figure 4.
(PDF)
