# Supplementary material for: phiC31 Integrase-Mediated Site-Specific Recombination in Barley
Source: PLoS One. 2012 Sep 14;7(9):e45353. doi: 10.1371/journal.pone.0045353 (PMC3443236; doi:10.1371/journal.pone.0045353)
Supplement: Figure S1 — Names and sequences of oligonucleotide primers used in this study. (PDF) [file pone.0045353.s001.pdf]

# - Figure S1 -

Primer	Nucleotide sequence (5' – 3')
gusFw	CCGGTTCGTTGGCAATACTC
gusRev	CGCAGCGTAATGCTCTACAC
gfpFw	GGTCACGAACTCCAGCAGGA
gfpRev	GACCACATGAAGCAGCACGA
gfpFw2	CGACCACTACCAGCAGAACA
gfpRev2	GAACTTCAGGGTCAGCTTGC
RecFw	TGACCAATTCCACAGTTTTCGCGAT
RecRev	GATCAGGAAGAGGGGAAAAGGGCACTA
C31IntFw	CCGACCACGAAGATTGAGGG
C31IntRev	GTTGCTTCCGGAAGTGCTTCC
F1actin	GAAGGCCTTCACTAGTGGCTCGAGGTCATTCAT
R1actin	TCCCCCCGGGGGGACTACAAAAAAGCTCCGCACGAGGCTGCA
F1attPgfp	CCCAAGCTTGTGCCCCAACTGGGGTAACCTTTGAGTTCTCTCAGTTGGGGGCGTAGGGCCA TGGTGAGCAAGGGCGAGGAGCTGTTCA
R1attBTnos	GGTGGTGACGTCGATGGGTGAGGTGGAGTACGCGCCCGGGGAGCCCAAGGGCACGCCCTG GCACCCGCACCGCGGCTTCGAG CGATCTAGTAACATAGATGACACCGCGCGCGA
