# Supplementary figures and images for: phiC31 Integrase-Mediated Site-Specific Recombination in Barley
Source: PLoS One. 2012 Sep 14;7(9):e45353. doi: 10.1371/journal.pone.0045353 (PMC3443236; doi:10.1371/journal.pone.0045353)

- Figure S2 -

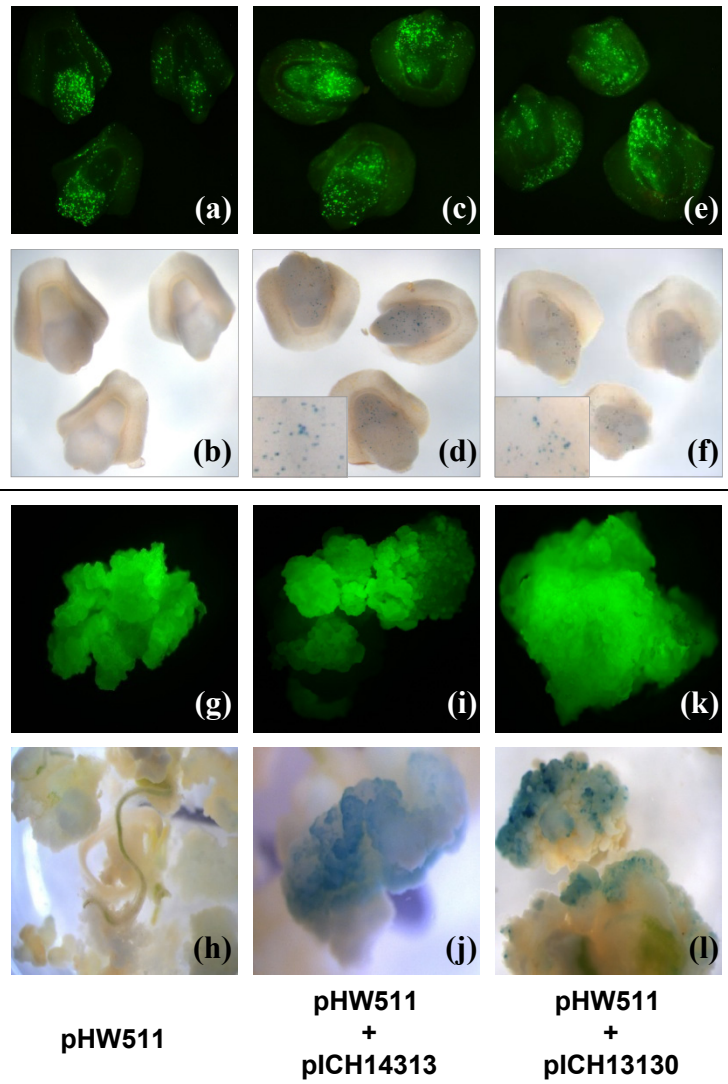

Supplement: Figure S2 — In vivo evaluation of vector constructs. (a–f) Analysis of barley embryos bombarded with the pHW511 plasmid (a, b) or co-bombarded with pHW511 and either pICH14313 (c, d) or pICH13130 (e, f). After 24 hours, the first GFP signals were detectable (Figure 2 a, c, e). β-Glucuronidase (GUS) staining of the embryos was performed 2 days after bombardment and revealed GUS signals in embryonic tissue that had been co-bombarded with both plasmids, pHW511 and an integrase vector, thereby indicating recombination (2 d, f). In contrast, in the control experiments carried out with pHW511 only, no GUS signal was observed (2 b). (g–l) Analysis of callus tissue derived from embryos that were bombarded with pHW511 (g, h) or co-bombarded with pHW511 and either pICH14313 (i, j) or pICH13130 (k, l). GUS expression was found in the majority of the embryo-derived calli that developed after co-transformation (j, l), but not in the calli of the control experiments (h). GFP expression was, as expected, ubiquitously present (2 g, i, k). (a, c, e, g, i, k) display fluorescence microscopy images; (b, d, f, h, j, l) present results obtained by β-glucuronidase (GUS)-staining. (PDF) [file pone.0045353.s002.pdf]

- Figure S3 -

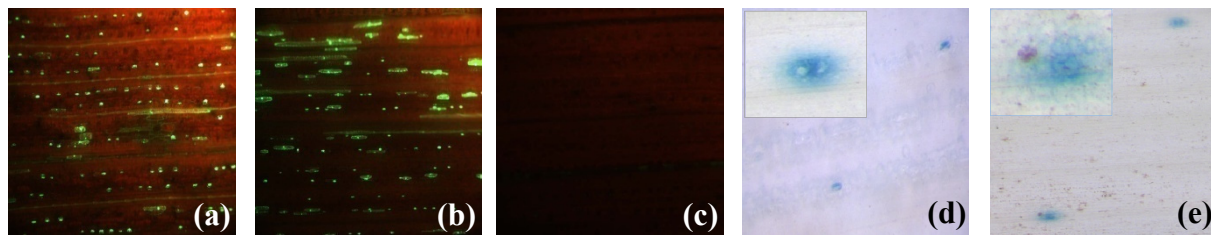

Supplement: Figure S3 — Transient assays for integrase activity. Bombardment of T0 plants harboring the integrase locus ICH13130 (a) or ICH14313 (b) with the viral vector pICH16710 which carries a GFP expression cassette and whose replication is activated after phiC31 integrase catalyzed recombination [14]. (c) Control experiment in which an untransformed plant was bombarded with pICH16710. (d, e) GUS staining was performed on leaf material of T0 plants harboring the integrase locus ICH13130 (d) or ICH14313 (e) after bombardment with the target vector pHW511. (PDF) [file pone.0045353.s003.pdf]

- Figure S4 -

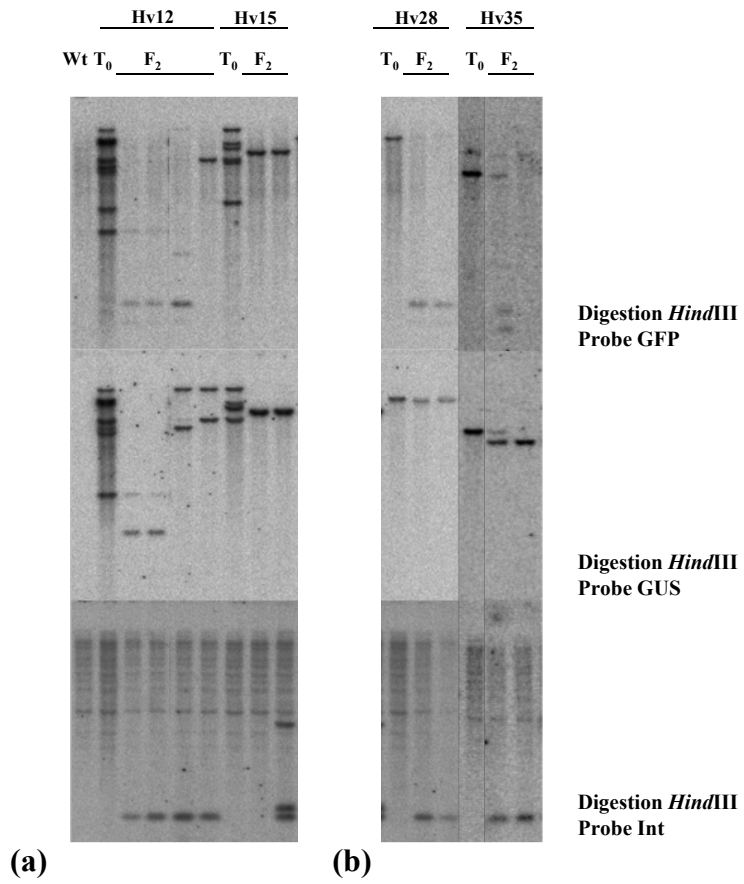

Supplement: Figure S4 — Resolution of complex loci through recombination. (a) Total DNA of plants containing multiple copies of the target locus was digested with the restriction enzyme HindIII and hybridized with probes GUS, GFP, and INT. This strategy allows for detection of a constant integrase-fragment and estimating of the copy-number of the target locus. Different patterns of F2 progeny plants can be explained by segregation of unlinked recombinant or non-recombinant loci, different outcomes of the recombination in the case of complex integration patterns or, recombination events that occur late in the development of the F1 plant and are independently inherited to the individual progeny plants. For comparison, the analysis of primary transformed plants carrying a single-copy of the locus HW511 are documented (b). (PDF) [file pone.0045353.s004.pdf]

- Figure S5 -

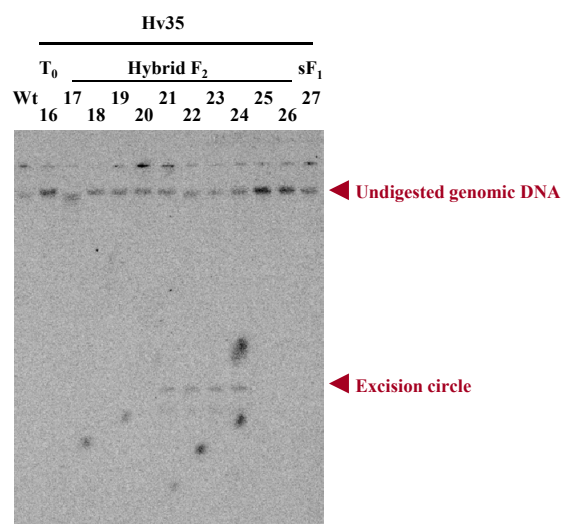

Supplement: Figure S5 — Detection of excision circles using undigested DNA. DNA gel blot analysis was carried out using undigested total DNA of transgenic plants containing a single copy of the target locus Hv35 and their descendants obtained by selfing (sF1) or crossing with transgenic plants carrying an integrase locus (hybrid F2). As controls, untransformed plants (Wt) are included. The membrane was hybridized with the probe GFP. The arrangement of plants is identical to that in Figure 4. (PDF) [file pone.0045353.s005.pdf]
